# Youth International Experience Is a Limited Predictor of Senior Success in Football: The Relationship Between U17, U19, and U21 Experience and Senior Elite Participation Across Nations and Playing Positions

**DOI:** 10.3389/fspor.2022.875530

**Published:** 2022-04-13

**Authors:** Henrik Herrebrøden, Christian Thue Bjørndal

**Affiliations:** ^1^RITMO Centre for Interdisciplinary Studies in Rhythm, Time and Motion, University of Oslo, Oslo, Norway; ^2^Department of Psychology, University of Oslo, Oslo, Norway; ^3^Department of Sport and Social Sciences, Norwegian School of Sport Sciences, Oslo, Norway; ^4^Norwegian Research Centre for Children and Youth Sports, Norwegian School of Sport Sciences, Oslo, Norway

**Keywords:** talent identification, talent development, athlete development, elite sport systems, youth sport

## Abstract

Athlete participation in youth international competitions is often regarded as crucial to the attainment of future success. However, the link between participation and performance in sports at youth levels and senior levels is unclear at best. To understand this relationship better we conducted two studies of male football players. In Study 1, we examined adult performance at the upper levels of football using a factor analysis and identified the characteristics that define what we termed a “Super Elite” level, which is the highest level of participation. This outcome measure was used in Study 2 to explore further the link between youth international experience and athletes' Super Elite experience. Overall, our results indicated that youth international experience is a limited predictor of participation at the Super Elite level of football. Participation at the U21 level was the strongest, most consistent predictor of Super Elite level participation. U17 participation was found to be either an insignificant or a negative predictor of subsequent participation in international football. The effect of U19 participation on later participation was partly significant, but weaker than the effect of U21 participation, and depended on the national context and the playing positions of the athletes. When looking at the effect of different youth career types, careers involving U21 international experience were the strongest predictors of later careers as Super Elite athletes. National governing bodies that want to ensure success in talent identification and development should therefore consider focusing fewer resources on youth international competitions in age categories before adulthood. A total of 1,482 players who had national football team experience at either the U17, U19, U21, or senior levels were included in our studies.

## Introduction

Sport organizations have focused increasingly on ways to identify and develop early athletic talent (Till and Baker, 2020). National governing bodies, for example, use considerable resources on talent identification and development programmes each year, including support for youth international competitions (Schroepf and Lames, 2018). How well a country's youth international teams (U-teams) perform is often seen as a key indicator of talent development productivity, and an indicator of likely future international success at the senior level. However, the relationship between youth sport performance and success in senior sport careers remains uncertain at best, both at an individual and team level (Johnston et al., 2018). This is complicated by the fact that talent identification and development systems vary between distinct sport cultures and between sport organizations (Andersen et al., 2015). Performance requirements, too, differ across playing positions and tactical formations (Gil et al., 2014; Modric et al., 2020).

Barreiros and Fonseca (2012) retrospectively examined the relationship between Portuguese elite athletes' involvement in international competitions in football, volleyball, swimming, and judo. The percentage of international senior athletes who had never participated in any international youth competition ranged from 6 to 44%, depending on the sport and the gender of the athletes. In team sports, particularly, a substantial number of international senior athletes had no international experience during their time as youth athletes. Barreiros et al. (2014) also prospectively examined athletes in those same sports across squads that were selected for international games. The overall results showed that only a third of athletes who participated at the international pre-junior level (U14/U15/U16) also participated as senior international players. In basketball, the best senior players in Europe were found not to have had more international youth experience compared to their lower-level counterparts (Kalén et al., 2017). In studies of athletics, only 17% of the male sprinters and 21% of the female sprinters included in the top-50 ranked athletes in the U18 category were able to reach a later ranking among the top 50 senior athletes in the sport (Boccia et al., 2020). Similarly, only 8% of male jumpers and 16% of female jumpers among the top-50 ranked athletes at the age of 16 years were able later to reach a top-50 senior ranking (Boccia et al., 2021a). Only 6 and 12% of male throwers and 16 and 24% of female throwers ranked in the top 50 athletes in the age categories of 16 and 18 years, respectively, were later able to achieve a similar top-50 status in senior elite athletics (Boccia et al., 2021b).

In contrast, Li et al. (2018) found that for junior athletes in combat sports, winning an international medal was a significant predictor of whether they would later win international senior medals. Similarly, Bjørndal et al. (2018) examined athlete progression in Norwegian handball national squads and showed that athletes who played in youth international competitions were later more frequently represented at the senior national team level, compared with athletes who had no youth international team experience. However, the *number* of match appearances in these instances was not associated with later success at the senior level.

Similar studies of German football suggest that approximately a third of youth international players (Güllich, 2014) to a half of youth international players (Schroepf and Lames, 2018) will become senior professionals. This suggests that there is a limited relationship between youth international experience and senior success in football. However, the predictive value of youth participation depends strongly on the type of youth international career in question. Player participation in the higher U-team age categories (for example, the U21 category) has been found to be a relatively successful indicator of later career achievements in senior football (Schroepf and Lames, 2018).

Overall, the findings we reviewed varied considerably by sport, gender, and by country. The inconsistencies and variations we found were, in part, due to discrepancies in the methodological approaches used by researchers (e.g., Boccia et al., 2020). Several studies, for instance, used descriptive statistical approaches (e.g., Barreiros et al., 2014) that lacked appropriate significance testing, and this made it difficult to measure effect sizes across the studies and across the different populations. Further, many of the sport studies were vaguely reported, which made it difficult to compare the findings accurately. Schroepf and Lames (Schroepf and Lames, 2018), for example, defined football success as “reaching [a] professional level in first or second Bundesliga as well as in first or second top European leagues” (p. 407). After reading this paper, we were unsure which specific European leagues had been included in their definition and measurement of professional status or player success. This was disappointing because the information was pertinent to our study.

The aim of our study was twofold. In Study 1, we sought to define the concept of senior elite participation and to create an outcome measure of participation at the highest levels of football using factor analysis. This defined outcome measure was then used as the basis for Study 2, in which we investigated the links between senior elite participation and international participation at the U17, U19 and U21 levels, across playing positions and nations. Our study was pre-registered on the Open Science Framework platform: https://osf.io/xd3rf/.

## Study 1: Defining Senior Elite Participation in Football

Before discussing what factors *predict* senior elite participation, it is important first to *define* what senior elite participation is. This is a challenging and complex process because of the different terminologies used in sport. Swann et al. (2015) note, for example, that football is a particularly competitive field and that all players from all top four tiers in England can be described as “professionals”. This, necessarily, makes the term inadequate as a precise classification of football elites. However, the level at which an athlete competes on a regular basis can still be regarded as the best indicator of a performance standard (Swann et al., 2015). In our present study, we therefore sought to identify the competitive categories that we could group together as an outcome measure of senior elite participation at the highest levels.

## Procedure, Data Analysis, and Results

Using online football databases, we identified 1,482 male football players from Denmark, Norway, Sweden, Belgium, Germany, and Portugal who had international football experience and gathered data on their participation in elite football. We then checked the trustworthiness of our data via a reliability test which is described in Appendix A. A full description of our participants and data collection is provided in Study 2.

Based on our reliability check, and because we were interested in football participation at the highest levels, we used player participation in the following events as potential options to define an outcome measure of *elite participation* in our study: (a) Senior international team matches; (b) the Champions League (CL); (c) the Europa League (EL); (d) the top five leagues in Europe; and (e) leagues ranked 6-10 in Europe. These competitive domains indicate a certain level of professional “success” in football but using each of them as separate outcome measures would have been analytically cumbersome. Instead, we hoped to make the process of hypothesis testing easier by reducing the outcome measures to one *Super Elite* factor that could be used as an outcome measure in this study. Hence, we took an exploratory approach to factor analysis based on participation data (i.e., how many appearances the recruited players had made in the selected competitive categories).

As shown in Table 1, most of the five variables we selected to define an elite player level in this study appeared to be significantly correlated. These correlations were subsequently confirmed to have a Kaiser-Meyer-Olkin value of 0.705, indicating sample adequacy, and a significant correlation value when tested with Bartlett's Test of Sphericity (*p* < 0.001). We therefore regarded the numeric outcome variables as well-suited to factor analysis.

**Table 1:** Pearson correlation between numbers of games in various categories of male elite football.

**Measure**	**1**	**2**	**3**	**4**	**5**
1. Sr. Ntl. Team	1	0.619^**^	0.129^**^	0.746^**^	0.512^**^
2. Top 5	0.619^**^	1	0.012	0.655^**^	0.523^**^
3. Rank 6-10	0.129^**^	0.012	1	0.134^**^	0.350^**^
4. CL	0.746^**^	0.655^**^	0.134^**^	1	0.416^**^
5. EL	0.512^**^	0.523^**^	0.350^**^	0.416^**^	1

** *Correlation is significant at the 0.01 level (2-tailed)*.

A principal components analysis (PCA) was deemed appropriate for our purpose of reducing the potential number of outcome measures in the current study (Jolliffe and Cadima, 2016). This analysis resulted in two components with an eigenvalue >1. The first component explained a substantial 56% of the variance, while the second explained 21.6%. We used Direct Oblimin rotation to allow for correlation among factors. The resulting solution suggested that four variables could potentially be loaded on the first factor. These variables were appearances in the CL, EL, Top five leagues, and a senior national team. To facilitate our interpretation of the components and to test their robustness as a single unit, a reliability analysis was conducted with these four variables as part of the same scale. The calculated Cronbach's alpha value of 0.48 was below the recommended cut-off value of .7. However, the output suggested a value of .77 if one item–namely, appearances in the Top five leagues–was deleted. We therefore decided to use the remaining three variables as part of the same factor to measure participation in elite football, namely: CL, EL and senior national team appearances. For the sake of completeness, we ran a reliability analysis on the remaining two factors (appearances in the top five leagues, and the leagues ranked 6 to 10, respectively). This provided further confirmation that they were too poorly related to be included as part of the same factor in this context.

To facilitate analysis and interpretation in our study, we decided to use the first factor from our factor analysis as the outcome measure for all our hypothesis testing. Inspired by Collins et al. (2016), we named our outcome component a ‘Super Elite' level, because we regarded international appearances at the senior level, CL, and EL as some of the most prestigious levels of male competitive football.

## Study 2: The Link Between Youth International Experience and Super Elite Participation

In Study 2, we sought to identify the predictors of participation at the senior elite level, both categorically and numerically. Specifically, we wanted to answer two research questions.

Our first question was: What predicts whether players will achieve Super Elite *status* (i.e., participation in one or more senior national team games, CL games and/or EL games, defined as “yes” or “no”)? To answer to this question, we used international participation in a U17, U19 and U21 team – both categorically (i.e., *status*, “yes” or “no”) and numerically (i.e., the number of games) – as our main predictor variables. Two relevant player characteristics were included so that we could examine how these interacted with the main predictors, namely player nationality and playing position. In addition to investigating the separate U-team predictors, we tested the effect of various U-team career types (i.e., combinations of U-teams represented) on Super Elite participation.

Our second question was: What predicts the number of Super Elite appearances players will achieve (i.e., the total number of games played in a senior international team, the Champions League and the Europa League) once they have reached this top level? This latter research question is related to our first research question, but at the same time they are distinct: a relatively substantial number of athletes may appear at the top levels at some point, but relatively few of these manage to achieve numerous appearances at the highest levels of competition (Güllich and Emrich, 2012). To answer our second question, we conducted subsample analyses by only including the players who had played one game or more at the Super Elite level (*n* = 482). Due to the more limited sample size in the Super Elite sample, we only used the main U-team variables (i.e., U17, U19, and U21 status and appearances) as our predictors of the number of games, without the inclusion of player position and nationality, and with no statistical test of U-team career types.

The following hypotheses related to the first research question, using our full sample: (H1a) A player's U17, U19 and U21 status will separately predict his Super Elite status, and the predictors will interact with nation cluster and playing positions; (H1b) A player's U17, U19 and U21 appearances will separately predict his Super Elite status, and the predictors will interact with nation cluster and playing positions; and (H1c) U-team careers that involve more than one U-team and/or U21 participation will be the strongest predictors of players' Super Elite status.

The following hypotheses related to the second research question, concerning the Super Elite players only: (H2a) U17, U19 and U21 status will separately predict the number of Super Elite appearances; (H2b) U17, U19 and U21 appearances will separately predict Super Elite appearances.

### Participants and Context

Our study sample included 1,482 male footballers born between 1990 and 1995. All players had a minimum of one official international appearance for their country. We recruited players from six different countries and grouped these into two clusters: (a) Scandinavian countries (Denmark, Norway, and Sweden); and (b) “Top nations” (Belgium, Germany, and Portugal–e.g., nations that were in the top 10 UEFA country coefficients list during 2020/2021 (https://www.uefa.com/nationalassociations/uefarankings/country/#/yr/2021).

The decision to include and group Scandinavian countries together into one nation cluster was driven by key contextual insights. Scandinavian nations share similar characteristics, including population size, social welfare state systems, and broad-based voluntary sport movements that serve as the basis for elite sport development (Andersen and Ronglan, 2012; Bjørndal et al., 2015). Our intention was therefore to compare Scandinavian countries, collectively, with more densely populated and highly-ranked football nations. The Top nations in our sample were chosen for pragmatic reasons: these countries had reliable national team statistics for individual players, and this information was available on their respective federations' web pages (see Appendix A for an elaboration on our process of checking data reliability and how this affected the recruitment process of our study). We were not able to obtain reliable national team statistics for other European Top nations (e.g., England, Spain).

The recruited players all had one or more appearances for the following national teams: Under 17 (U17), Under 19 (U19), Under 21 (U21), and/or Senior.

Our recruitment was based on these team categories for several reasons. First, official tournaments (such as the UEFA European Championships) are hosted for these defined age group categories. Being selected to these teams is therefore prestigious and the likelihood of finding reliable data online was assumed to be high. Second, other U-team configurations are used and prioritized differently across nations. Denmark, for example, has no U15 team, and Sweden has no U20 team. Hence, we were interested specifically in team categories that would facilitate cross-nation comparisons.

We ran *a priori* power analyses using the statistical software G^*^Power to ensure that our sample included enough players in the least frequent positions (e.g., goalkeepers). Different scenarios were used for our power calculations because of the diversity of our analyses. Based on our power analyses, we sought to include a minimum of 90 players for each position. We also wanted to focus on footballers who were old enough to have had the opportunity to achieve success at the international senior level. Data were therefore collected for male players born between 1990 and 1995 as this was expected to provide us with a sufficiently large total sample size, and the required minimum of 90 players in each position.

### Procedure

**Figure 1:**
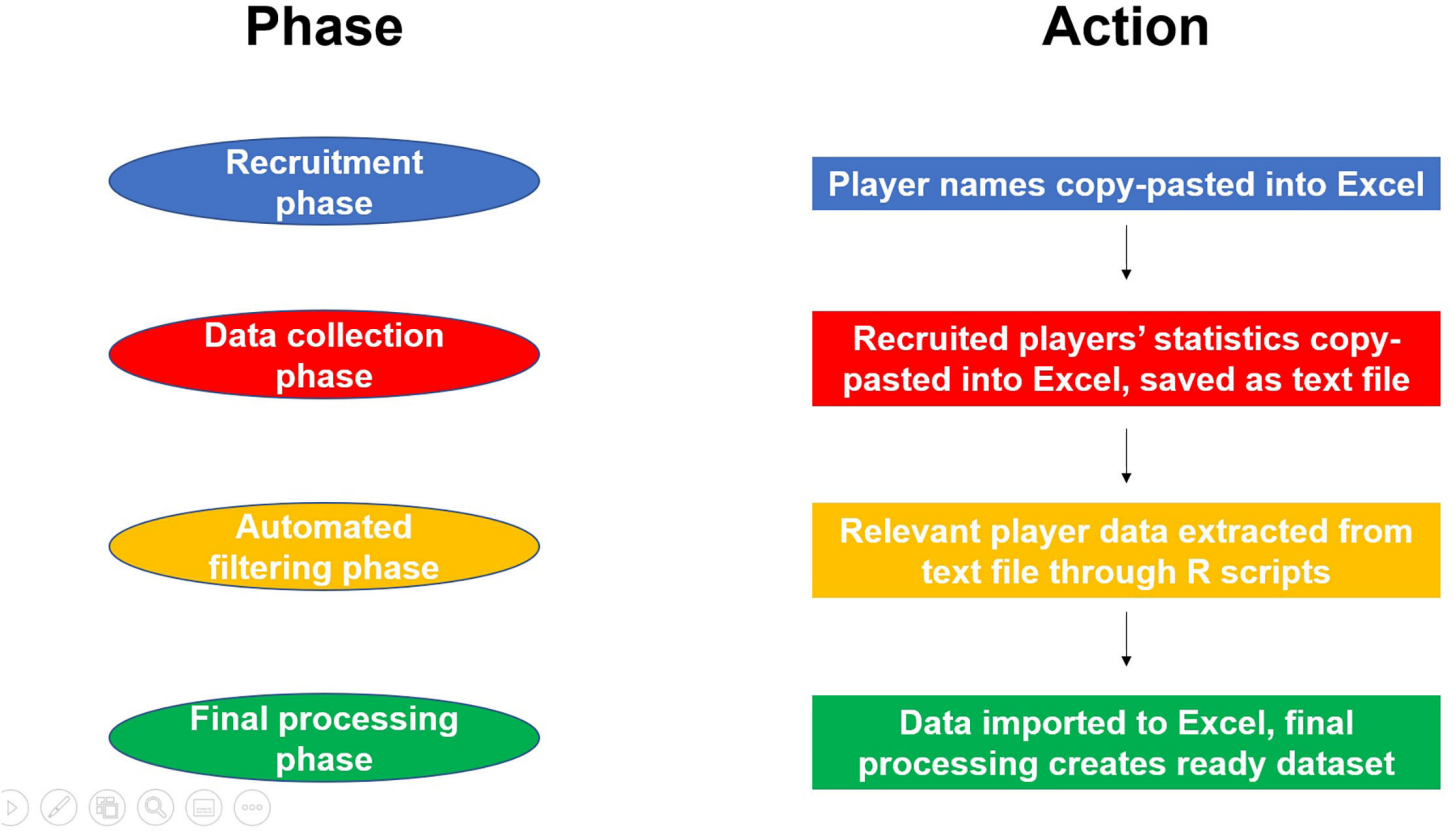
A flow chart showing our data work in four different phases, including actions made in each phase.

Figure 1 shows a simplified graphic of our four-stage workflow process. Each of the steps is described in detail below.

#### Recruitment

We identified U-team squads that played in the year range 2005 to 2017. This recruitment process for our study was primarily done via Transfermarkt (TM; www.transfermarkt.com), a website which provides extensive football-related information and statistics. We searched the site for the respective team categories (e.g., “Germany U17”) and selected the squad lists for the appropriate years. The TM website did not have squad information for every cohort in every U-team, especially for teams in the Scandinavian countries. We therefore searched for data for nations' U-team appearances on national football federation websites to find supplementary squad information. In the rare instances in which the TM website and/or federation websites did not include useful squad lists for our years of interest, we used a third website (www.besoccer.com) to search for U-team appearances and squad information.

After finding information about the U-team players, we then searched for players who had represented their nation's senior team but had not made any U-team appearances. This was done using an “Advanced player search” on the TM website, using the appropriate citizenship and birth year of the players. The following alternatives were then selected from the TM website's menu: (a) “Current national team player”: (b) “Former (not current) national team player”; and (c) “Retired from national team”. This search identified additional players who had not already been included in our initial searches, and these were then added to the study sample. After filtering out players who were born outside our intended age range (1990–1995), we then created an Excel sheet with a list of players and moved to the next stage of collecting player data.

#### Data Collection

Individual player information was copied and pasted manually from the TM website's player profiles into our Excel list of recruited players. The comprehensive list of information we retrieved from the TM website was added to our study pre-registration form, and this can be accessed on the Open Science Framework platform (https://osf.io/xd3rf/). For the purposes of both our studies, our particular focus was on: (a) basic player characteristics (e.g., birthdate, playing position); (b) appearances for national teams; (c) appearances in the Champions League, the Europa League, as well as top tiers in the 15 highest-ranked nations on the UEFA country coefficients list for 2020/2021.

We were unable to find a TM profile for 27 of the study's recruited players. In these instances, we searched for information about the players via Google and several online football databases (such as www.altomfotball.no, and www.playmakerstats.com). The results of these searches confirmed our assumption that these players had likely never played at the highest levels of football or in any of the top European leagues. While searching for these players' names online, we found evidence that they were within the appropriate age range. We felt certain therefore (beyond a reasonable doubt) that these players had gained international U-team level experience but lacked experience in senior professional football, and that they should be included in our analyses.

Information from player profiles on the respective football federation web pages were also copied into our Excel sheets, so that official national team statistics were included for each player.

#### Automated Data Filtering

The raw data we gathered from the TM profiles and from the federation web sites were saved as CSV text files and run through scripts in the statistical program R. One script was used per nation, and the code can be found at the GitHub platform: https://github.com/henrher/Youth-and-senior-success-in-soccer/find/main. The data set and a detailed description of our procedure are also publicly available, at the OSF website: https://osf.io/xd3rf/.

#### Final Data Processing

After running the initial script on the raw data, some manual corrections were needed. Our scripts, for example, were unable to retrieve player appearances in the correct way for all players, especially in particular leagues (e.g., the Portuguese top tier, which had recently changed its name). Several duplicate records had to be removed, and we also removed players who: (a) had appeared in games for more than one nation during their career, according to the TM website; and (b) had 0 appearances for the national teams of interest (even though they had been drafted), according to their federation website profiles. Most of the changes to the raw data set were implemented manually in Excel. For certain issues, we ran the raw data through modified scripts in R to retrieve the information we needed. Instructions for how to collect data using our approach can also be found at the OSF website: https://osf.io/xd3rf/.

After this data processing, the total sample of 1,482 players were relatively well distributed across the selected nations and playing positions (see Table 2 below). A total of 716 players were selected from the Scandinavian countries, and 766 were from the Top nations. The process of categorizing footballers' playing positions–in other words, of deciding which positions could be put under appropriate “umbrella terms” –can be done in several different ways. In our study, we used the same playing position categories described by Kalén et al. (2019). To clarify and document how we operationalized these playing positions, we have included a parenthesized list showing which original playing positions (as listed on the TM profiles, under players' “Main position”) were used when deciding on our final position categorizations. Eighty four players were not listed with a specific playing position on TM. These players were included in the analyses as part of their own (unspecified) positional category, but we decided not to focus on or report any effects involving this category in our Results because our focus was on the specified position categories listed in the Table 2.

**Table 2:** The nationalities and playing positions included in our study.

**Nations**	**Playing positions**
Denmark (*n* = 238)	Goalkeeper (Goalkeeper; *n* = 152)
Norway (*n* = 250)	Center-back (Center-back; *n* = 244)
Sweden (*n* = 228)	Full-back (Left-back, Right-back; *n* = 252)
Belgium (*n* = 243)	Central midfield (Central midfield, Defensive midfield, Attacking midfield, Left midfield, Right midfield; *n* = 402)
Germany (*n* = 301)	Winger (Left winger, Right winger; *n* = 157)
Portugal (*n* = 222)	Striker (Center-forward, Second striker; *n* = 191)

### Data Analysis

For our main analyses of the links between the various Super Elite and U-team variables, we planned to apply a variety of standard regression models. However, it was evident that our data did not meet many of the common assumptions associated with such statistical approaches, and that additional measures would be required.

The first issue became evident when we conducted a binary logistic regression analysis to test Hypothesis 1b (namely, the effect of numerical predictors (U-team appearances), along with fixed factors (nationality and playing position), on athletes' Super Elite status). This model's results were difficult to interpret for two reasons. First, the calculated Hosmer-Lemeshow test value was highly significant (*p* < 0.001), indicating that there was a poor fit between the model we were using and our data. Second, the odds ratio values [Exp(B)] were less intuitive when they were based on continuous predictor variables, as opposed to categorical predictor variables (as used in our test of Hypothesis 1a). We therefore recoded our continuous predictor variables into a nominal variable with limited categories. Specifically, we labeled players according to the following classification for each U-team: (a) Category 0: 0 games; Category 1: 1–5 games; Category 2: 6–10 games; Category 3: 11 games or more. Doing so allowed us to run a logistic regression analysis without violating the Hosmer-Lemeshow test assumptions. This variable coding was therefore used when testing our numerical U-team variables as predictors of Super Elite status.

In our subsample analysis, with numerical variables only (i.e., U-team appearances as predictors of Super Elite appearances), the data appeared to be non-normally distributed. Specifically, *heteroscedasticity* was indicated by a scatterplot and confirmed by statistical tests of this phenomenon in IBM SPSS Statistics v27 (e.g., White's Test, *p* < 0.001). We therefore decided not to use regular multiple regression analysis, and substituted this method instead with general linear models using *robust standard errors* (Hayes and Cai, 2007) when testing the effect of U-team participation on Super Elite appearances.

An Alpha value of 0.01 was our cut-off point for statistically significant effects. We used partial eta squared (η*p*^2^) as our measure of effect sizes in general linear models, and we considered.01, 0.06 and 0.14 as values indicating small, medium and large effects, respectively (Richardson, 2010). All statistical analyses were conducted using the IBM SPSS Statistics v27 software.

## Results

### Part 1: What Predicts a Player's Super Elite Status?

We conducted a logistic regression analysis of the players' U-team status (whether a player had represented a U17, U19, or U21 team, respectively), as well as fixed factors (national cluster and playing position), as the independent variables. Interactions between the U-team predictors and fixed factors were also considered in the regression model. The full model was found to be statistically significant, χ^2^ (*df* = 30, *n* = 1,482) = 542.029, *p* < 0.001. This suggested that the model allowed us to distinguish between players with, and without, Super Elite football experience. The model explained between 29.8 and 41.6% of players' Super Elite variance, measured by a Cox and Snell R square value and Nagelkerke R Squared value respectively. 79.8% of cases were found to be correctly classified. Table 3 provides an overview of the U-team variables' effects.

**Table 3:** U-team status effects on Super Elite status.

**x**	**B**	***SE***	**Sig**.	**Exp(B)**	Lower CI^*^
U17	0.084	0.287	0.771	1.087	0.619
U19	1.023	0.313	0.001	2.781	1.506
U21	2.417	0.281	<0.001	11.212	6.466

* *95% confidence interval for Exp(B), lower limit*.

U19 and U21 status were the only significant predictors of players' later Super Elite status. However, the effects of these two predictors were of different orders of magnitude. The odds ratio values [Exp(B)] provided useful indicators of the probability of a player achieving Super Elite experience when he went from Category 0 (having not played for the given U-team) to Category 1 (having played for the given U-team) in these dichotomous U-team variables. A player was therefore 2.78 times more likely to obtain Super Elite level experience if he had represented his country at the U19 level than if he had not. If a player had represented his U21 side, he was then 11.21 times more likely to have Super Elite experience compared to those players who had not.

U19 status showed a significant interaction with nation cluster (*p* = 0.006). We therefore ran one regression model per cluster, using the dichotomous U-team predictors as independent variables. Our findings suggested that U19 status is a significant predictor of Super Elite status in the Top nations [Exp(B) = 2.244, *p* < 0.001], but not in Scandinavia [Exp(B) = 1.009, *p* = 0.969]. In other words, U21 status was a significant predictor throughout the sample, while U19 status depended on the particular nation cluster when predicting who would reach Super Elite status. It is, however, worth noting that the Hosmer-Lemeshow values we calculated were significant (*p* < 0.001) for both these follow-up regression models, and that caution is generally needed when interpreting analyses on sub-samples. Playing position was found not to be significantly related to Super Elite status as a main effect or in interaction with the predictors.

**Figure 2:**
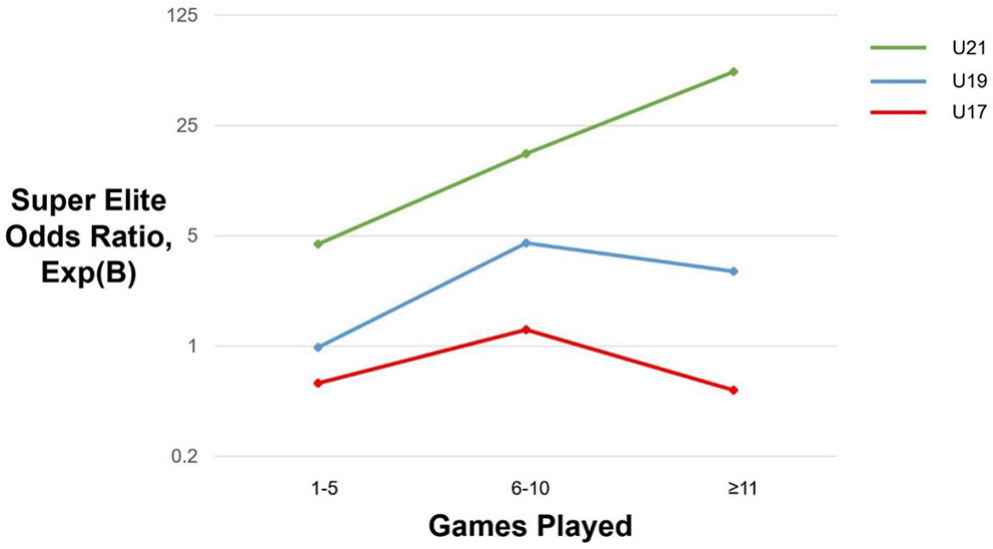
Likelihood of achieving Super Elite status as a function of the number of games for various U-teams. Please note that the values on the Y-axis are nonlinear. We have used a logarithmic scale (of increasing magnitude) to reveal the nuances at the lower end of the graph and to capture the trajectory of the U21 odds ratio values toward the higher end of the spectrum.

Next, we conducted a binary logistic regression analysis using players' U-team appearances as numerical predictors along with the fixed factors as independent variables. Interactions between the main predictors and fixed factors were again investigated. As discussed in the Methods (Data Analysis) section of this paper, each player's number of appearances was coded into four categories (0, 1–5, 6–10, 11 or more, respectively) in each U-team, for methodological reasons. The full model was found to be statistically significant, χ^2^ (*df* = 75, *n* = 1,482) = 672.661, *p* < 0.001. The respective Cox and Snell R square value and Nagelkerke R Squared value suggested that the model explained between 36.5% and 50.9% of variance in Super Elite status in our sample. 82.2% of cases were correctly classified. Of the main predictors, the U19 (*p* < 0.001) and U21 (*p* < 0.001) variables, and not U17 appearances (*p* = 0.191), were found to have a significant impact overall on a player's Super Elite status. When exploring categories within the U-teams, interesting nuances were observed. Figure 2 visualizes changes in the odds ratio [Exp(B)] when players moved from Category 0 to 1, from Category 1 to 2, and from Category 2 to 3 in the various U-teams. Any value above the line drawn where the Exp(B) value equals 1 indicates the likelihood of a player reaching the Super Elite level would *increase*, while any Exp(B) value between 0 and 1 (i.e., below the line) indicates that the probability of a player reaching the Super Elite level would *decrease* when entering a given category.

As shown in the graph, the U19 pattern shows an increase in the likelihood of players attaining Super Elite status when they move from Category 1 (1–5 games) to Category 2 (6–10 games). This likelihood decreases again when players enter Category 3. Only the second U19 category had a significant ability to predict a player's Super Elite status (Exp(B) = 4.484, *p* < 0.001). The visual pattern was similar for players in the U17 categories, albeit with odds ratio values that were lower–some even below 1–suggesting negative predictive values. No statistical significance was found for any of the U17 categories. The development within the U21 variable, on the other hand, more closely resembled exponential growth, as players moved into Category 1 [Exp(B) = 4.425, *p* < 0.001], Category 2 [Exp(B) = 16.629, *p* < 0.001], and Category 3 [Exp(B) = 54.664, *p* < 0.001].

The abovementioned U19, Category 2 (6–10 games) showed a significant interaction with nation cluster (B = −1.151, *p* = 0.10, Exp(B) = 0.316). This finding was intriguing because Category 2 was the only significant U19 category found in the main effects. We therefore ran one regression model per nation cluster, using the U-team predictors as independent variables. The results echoed our previous sub-sample analyses. Specifically, only the U21 category, at all category levels, was found to be a significant predictor of whether Scandinavian footballers achieved Super Elite status. On the other hand, significant effects of appearances at the U19 level [all categories except for Category 1 (1–5 games)] and U21 (all categories) were found in the model of Top nations. Hosmer-Lemeshow values were significant at the conventional level (*p* < 0.05) for both these follow-up regression models.

Additionally, the U19 Category 2 (6–10 games) showed a significant interaction with the goalkeeper position (B = −2.589, *p* = 0.007, Exp(B) = 0.075). This position was also noticeable because of interaction trends found with other U-team categories that would have been significant at a conventional level of significance (*p* < 0.05). Hence, we decided to run a regression model using only the 152 goalkeepers in this sample, using their U-team appearances as predictors. Although this test had a limited sample size and should be interpreted with caution, the results suggested that only U21 appearances (all categories) were significant at the level of *p* < 0.01 and had a positive increase in predictive value in each category. In summary, the number of U19 appearances did not significantly predict Super Elite status among Scandinavian players and among goalkeepers.

Finally, we explored the effect of different U-team career types on athletes' Super Elite status. We divided our player sample into all possible national team career combinations using eight categories (see Table 4 below). To test the predictive value of each career type, a binary logistic regression model was employed. Descriptive statistics showed that the most common career type of the selected players was “U17 only”. This category was therefore used as a reference group, and this enabled us to test the effects of increasing career length and/or latency by comparing all other career types with those who only had U17 experience.

**Table 4:** U-team types of careers in relation to Super Elite status.

**Career Type**	***N***	**B**	***SE***	**Sig**.	**Exp(B)**	Lower CI^*^
U17+U19+U21	293	3.860	0.286	<0.001	47.464	27.096
U19+U21	151	3.600	0.307	<0.001	36.600	20.043
U21-only	129	3.030	0.311	<0.001	20.693	11.254
U17+U21	30	3.014	0.446	<0.001	20.375	8.501
U17+U19	276	1.052	0.315	0.001	2.863	1.544
U19-only	236	0.881	0.332	0.008	2.414	1.259
Senior-only	25	24.217	8,038.594	0.998	3.292E+10	<0.001
U17-only	342	N/A	N/A	N/A	N/A	N/A

* *95% confidence interval for Exp(B), lower limit*.

The model was statistically significant, χ^2^ (*df* = 7*, n* = 1,482) = 599.336, *p* < 0.001. This suggested that the model was able to distinguish between players with, and without, Super Elite football experience, based on their career type. The model explained between 33.3 and 46.4% of variance in the players' Super Elite status, as measured by a calculated Cox and Snell R square residual and a Nagelkerke R Squared value respectively. 80.0% of our cases were found to be correctly classified.

All the categories were statistically significant except for the player group with senior national team experience only (*n* = 25). The table below provides an overview of the career types in relation to players' Super Elite status. The table is sorted in descending order, with the significant career types and the largest odds ratio values on top.

The calculated odds ratio values indicated that there were substantial differences between the player career types that involved U21 participation on one hand, and those that had no U21 participation on the other. Representation at the U21 level, especially in combination with selection to an U19 team or both the other U-teams, was found to be indicative of players having a dramatically increased chance of achieving Super Elite status.

### Follow-Up Analysis: Does Earlier U-Team Status Predict Later U-Team Status?

Our primary concern in this study was the link between U-team participation and a player's Super Elite participation. However, since a U21 status, and to some extent a U19 status, appeared to significantly predict a player's later Super Elite status, we decided to conduct binary logistic regression analyses to explore the relationships between the categorical U-team variables. Our first regression model tested whether U21 status could be predicted by a player's U17 or U19 status. Our results suggested that a player's U17 status was a negative and significant predictor [Exp(B) =0.515, *p* < 0.001] while his U19 status was a positive and significant predictor [Exp(B) = 1.885, *p* <0.001] of U21 experience. U17 status was also a negative and significant predictor of U19 status [Exp(B) = 0.609, *p* <0.001] in our subsequent regression model. In summary, players in our sample who had U17 experience were less likely to gain experience at the U19 and U21 level, compared to those players with no U17 experience. Participation in one or more U19 games, on the other hand, was a significant predictor of participation in one or more U21 games. The Hosmer-Lemeshow test value for our first binary regression model was significant, but the significance and direction of the relationships between U-teams were confirmed by follow-up Chi square tests.

### Part 2: What Predicts the Number of Appearances at the Super Elite Level?

Only players with Super Elite experience (*n* = 482) were included in the analyses targeting this question. We first tested the predictive ability of U-team status in relation to Super Elite appearances. A univariate regression model with robust standard errors [HC3 method; see Long and Ervin, 2000] was employed. The adjusted R squared value we calculated was 0.017, which suggested that our model explained 1.7% of the variance in the number of Super Elite appearances players had. The effect of their U17 status (B = 2.922, *p* = 0.322, η*p*^2^ = 0.002) and U19 status (B = −3.728, *p* = 0.267, η*p*^2^ = 0.003) was found not to be significant. Conversely, a significant main effect was found for U21 status (B = −10.204, *p* = 0.003, η*p*^2^ = 0.019).

Next, a univariate regression model with robust standard errors (HC3 method) was conducted to test the effects of U-team appearances on Super Elite appearances. The calculated adjusted R squared value was .032, which suggested that our model explained 3.2% of variance in the number of Super Elite appearances players achieved. Effects of U17 appearances (B = 0.620, *p* = 0.207, η*p*^2^ = 0.003) and U19 appearances (B = 0.048, *p* = 0.878, η*p*^2^ < 0.001) were not significant, while U21 appearances (B = 0.506, *p* = 0.003, η*p*^2^ = 0.018) was a significant predictor of Super Elite appearances.

Overall, our two subsample analyses yielded highly similar results, suggesting small yet significant and positive effects of U21 participation on Super Elite appearances.

## Discussion

Our study was designed to determine whether youth international experience was a predictor of player participation at the highest level of football, which we termed the Super Elite level. In Study 1, we used factor analysis to define this highest level of participation as a Super Elite participation factor–namely, player participation in senior international squads, the Champions League, and/or the Europa League. In Study 2, we tested hypotheses concerning whether player participation in U17, U19, and U21 teams were associated with Super Elite status across nationalities and playing positions. We also tested the predictive ability of U-teams in relation to Super-Elite appearances, once players had reached Super Elite status. Overall, our study extends the existing literature that suggests that performance in youth categories is a limited indicator of senior success.

Our first study contributes to the challenge of defining elite performance levels in football (Swann et al., 2015). The proposed Super Elite factor provided us with a clear outcome measure, and it may be used or built upon in future studies on higher levels of elite football. In our second study, the combinations of categorical and continuous variables further enabled us to draw nuanced conclusions. We were able to explain far more variance in Super Elite status than we were able to predict the number of games at the highest level. Specifically, our models that tested who reached the Super Elite category explained up to 50.9% of variance, while models predicting the number of Super Elite games explained only 3.2% of variance at the most. This speaks to the difficulty of predicting which young players will end up with numerous appearances at the top level of football (Güllich and Emrich, 2012).

We found several variables that significantly predicted players' status (i.e., one or more games) in Super Elite football: (a) U21 status and U21 appearances (number of games); (b) U19 status as long as the players were from the Top nations, and U19 appearances if the players were from the Top nations or played outfield positions (i.e., not goalkeepers); and (c) all careers involving a U-team, except for those with only U17 experience – particularly ‘late' careers (i.e., involving U21 experience) and especially ‘late and long' careers (i.e., experience from U21 combined with one or more U-teams).

Unsurprisingly, the U21 variables were found to be the most significant, consistent, and substantial individual predictors of Super Elite status, and the only significant predictor of Super Elite appearances. This suggests that U21 participation is an indicator of senior success in football. In contrast, U17 participation showed no significant relationship to Super Elite participation. This is a noteworthy finding given that U17 selection is regarded as prestigious in football and is seen as important by football federations and the media alike. As for U19 participation, its role as a predictor may be summarized as “it depends”: the roles of U19 status and appearances appeared to depend on player characteristics, namely their nationality and their playing position, and this we will discuss later. Overall, our results echo previous findings reported by Bjørndal et al. (2018) and Schroepf and Lames (2018), which suggest that indicators of senior sporting success appear later rather than earlier in athletes' careers.

Inspired by the approach of Schroepf and Lames (2018), we explored the predictive value of different U-team career types. When closely examining the descriptive statistics, two career types were found to be uncommon in our sample of 1482 players. First, the combination of U17 and U21 participation, without any U19 participation in-between, was rare (*n* = 30). This finding was similar to those reported in past studies in football that reported substantial player turnover and few “comebacks” after de-selection from U-teams (Güllich, 2014; Schroepf and Lames, 2018). The second small and uncommon group consisted of players who had no U-team appearances, and only senior national team experience (*n* = 25). This finding was similar to those reported by Bjørndal et al. (2018) in the context of handball.

Nonetheless, our study showed that it is possible for players to reach the Super Elite level even if they have had no appearances in youth international teams. Schroepf and Lames (2018) reported that all 37 German senior national team players in their study, born between 1987 and 1994, had represented a U-team. Unexpectedly, in our study we were able to identify six German players who had no international youth experience (for any U-team, ranging from U15 to U23), but who had represented the German senior national team (Jonas Hector, André Hahn, Diego Demme, Marcel Halstenberg, Mark Uth, and Robin Gosens–all born in the period 1990–1994). Why these players were not included in the Schroepf and Lames (2018) analyses is still unclear, even after a thorough reading of their paper. This underlines the imperative of openness and clarity in study reporting. However, our overall findings were similar to those of Schroepf and Lames (2018) who observed that later selection and longer careers in youth international teams are indicators of successful senior football careers, although in their study they included all U-teams from U15 through U21 while we focused on the U17, U19, and U21 categories only.

To our knowledge, ours is the first study of its kind to include playing position as a variable, and our results need to be tested and replicated before generalizations can be made with confidence. In terms of effect sizes, the interactions we found between U-team predictors and positions were nuanced and small. Nonetheless, we found in our sample that one certain predictor effect disappeared when analyzing goalkeepers only (*n* = 152). Specifically, U19 appearances did not predict the later Super Elite status for goalkeepers. One possible reason for this is that the U-team selection of goalkeepers may strongly reward growth and maturation, due to the anthropometric demands of the position (Deprez et al., 2015; Brustio et al., 2018), rather than simply the skills that help players gain a successful senior career. Further studies are needed to replicate and elaborate on the relationship between playing positions and career transitions in team sports.

Our findings suggest that the link between U-team participation and senior elite participation is stronger in top-ranked nations compared to the Scandinavian countries. When analyzing our Scandinavian recruited players only, we found that U19 participation–categorically and numerically – was unable to significantly predict who will end up in Super Elite football, while the opposite was true when analyzing players from Belgium, Germany, and Portugal. This may be due to the differences between how sport, in general, and talent identification and development in particular, are organized across national contexts. For example, in Scandinavian countries, the voluntary-based sport model is characterized by decentralized, egalitarian structure with low levels of professionalization (Ibsen and Seippel, 2010). Compared to most academy-based programmes in other countries, this model represents a clear point of difference (Bjørndal et al., 2015). The less structured and non-commercial Scandinavian model may increase overall sports participation initially, and the length of playing careers eventually, and therefore weaken the association between formal talent identification programmes and senior success (Andersen et al., 2015). In comparison, the elite sport systems of Belgium, Germany and Portugal may inflate the effects of selection mechanisms at an earlier age, compared to the Scandinavian countries, resulting in stronger associations between formal talent identification and development programmes and senior success. Exploring these assumptions further opens an important new line of inquiry for future research.

Despite the strengths of this study, some limitations should be considered before these findings can be generalized across populations. Firstly, our sample consisted of international players only. Hence, the career pathways of successful senior elite players who do not have any competitive experience from youth international teams should be of particular interest in future research. Secondly, we included only U17, U19 and U21 teams as our main predictors for methodological reasons. It is worth noting that some countries, such as Norway, place heavy emphasis on other U-team classifications as well (e.g., U18, U20). It is therefore unclear whether including more teams, as has been done in other studies (Güllich, 2014; Schroepf and Lames, 2018), may lead to different results. However, our aim was to investigate teams that were comparable between nations, and our methodological decision facilitated this purpose.

## Conclusion

The study demonstrates that competitive experience gained from youth international teams is a limited predictor of senior success in professional football, and that there were few distinct variations to this across nationalities and playing positions. Participation at the U21 level was the strongest, most consistent predictor of Super Elite level participation. U17 participation was found to be either an insignificant or a negative predictor of subsequent participation in international football. The link between U19 participation and later participation was partly significant, but weaker than participation at the U21 level, and depended on national context and playing position. When looking at the effect of different youth career types on later participation, careers that included U21 international experience were the most substantial predictors of Super Elite careers.

Considering the nonlinear nature of development in sport and how performance before adulthood is strongly influenced by growth and maturation, systematic talent identification is limited at best. Investments in association-based youth talent identification and selection systems in football may not be useful because such participation does not appear to be a strong predictor of later international elite football participation. Our findings indicate that sport governing bodies need to re-consider their strategies for talent identification and development: before players reach adulthood, fewer resources could be spent on helping a limited number of selected players gain competitive international team experience. This may mean, instead, that the limited economic and human resources that are available should be re-allocated to more local activities that promote recruitment, participation, and development at the grassroots level. Doing so could achieve broader benefits without compromising the development of elite sport.

## Data Availability Statement

The datasets presented in this study can be found in online repositories. The names of the repository/repositories and accession number(s) can be found below: https://osf.io/xd3rf/.

## Ethics Statement

Ethical review and approval was not required for the study on human participants in accordance with the local legislation and institutional requirements. Written informed consent from the participants' legal guardian/next of kin was not required to participate in this study in accordance with the national legislation and the institutional requirements.

## Author Contributions

Both authors listed have made a substantial, direct, and intellectual contribution to the work and approved it for publication.

## Conflict of Interest

The authors declare that the research was conducted in the absence of any commercial or financial relationships that could be construed as a potential conflict of interest.

## Publisher's Note

All claims expressed in this article are solely those of the authors and do not necessarily represent those of their affiliated organizations, or those of the publisher, the editors and the reviewers. Any product that may be evaluated in this article, or claim that may be made by its manufacturer, is not guaranteed or endorsed by the publisher.
