# Corrigendum: Youth International Experience Is a Limited Predictor of Senior Success in Football: The Relationship Between U17, U19, and U21 Experience and Senior Elite Participation Across Nations and Playing Positions

**DOI:** 10.3389/fspor.2022.954943

**Published:** 2022-06-29

**Authors:** Henrik Herrebrøden, Christian Thue Bjørndal

**Affiliations:** ^1^RITMO Centre for Interdisciplinary Studies in Rhythm, Time and Motion, University of Oslo, Oslo, Norway; ^2^Department of Psychology, University of Oslo, Oslo, Norway; ^3^Department of Sport and Social Sciences, Norwegian School of Sport Sciences, Oslo, Norway; ^4^Norwegian Research Centre for Children and Youth Sports, Norwegian School of Sport Sciences, Oslo, Norway

**Keywords:** talent identification, talent development, athlete development, elite sport systems, youth sport

In the published article, there was an error in Figure 2 as published. In Figure 2, we provided the wrong label for the category on the far right of the x-axis. Specifically, the label said “11–15.” The correct label is “≥11.” The corrected Figure 2 and its caption appear below.

**Figure 2:**
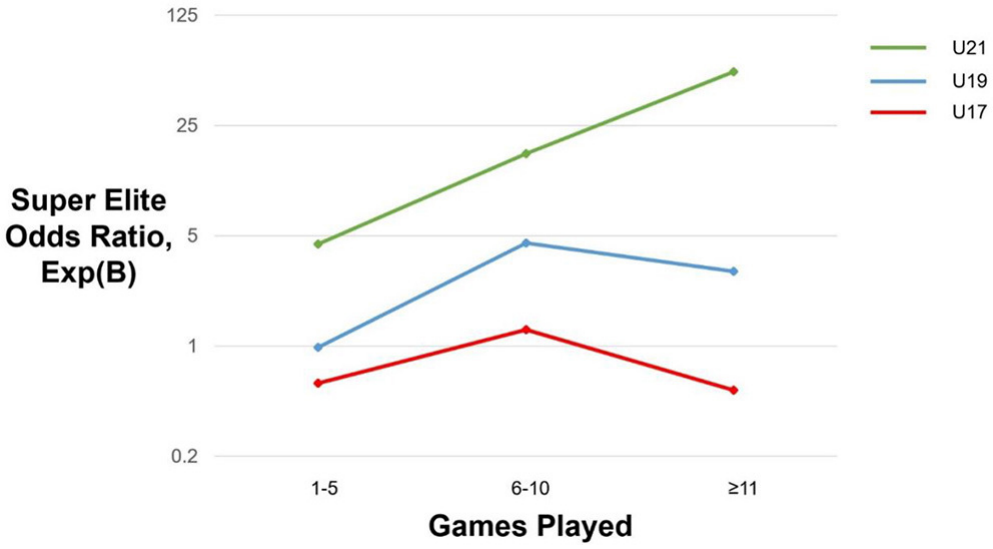
Likelihood of achieving Super Elite status as a function of the number of games for various U-teams. Please note that the values on the Y-axis are nonlinear. We have used a logarithmic scale (of increasing magnitude) to reveal the nuances at the lower end of the graph and to capture the trajectory of the U21 odds ratio values toward the higher end of the spectrum.

The authors apologize for this error and state that this does not change the scientific conclusions of the article in any way. The original article has been updated.

## Publisher's Note

All claims expressed in this article are solely those of the authors and do not necessarily represent those of their affiliated organizations, or those of the publisher, the editors and the reviewers. Any product that may be evaluated in this article, or claim that may be made by its manufacturer, is not guaranteed or endorsed by the publisher.

