# Supplementary material for: Youth International Experience Is a Limited Predictor of Senior Success in Football: The Relationship Between U17, U19, and U21 Experience and Senior Elite Participation Across Nations and Playing Positions
Source: Front Sports Act Living. 2022 Apr 13;4:875530. doi: 10.3389/fspor.2022.875530 (PMC9044071; doi:10.3389/fspor.2022.875530)
Supplement: Supplementary file 1 [file Data_Sheet_1.docx]

**Appendix A**

**Reliability test of data**

We report on the accuracy of Transfermarkt (TM) data regarding player appearances in international games and leagues. This is done to inform readers of our paper and other scholars who are planning to use the TM website in future research. Before we present our findings, we add a note of caution, emphasizing that this reliability check we conducted was not something that we can claim to be either complete or definitive, since we checked only a limited number of players in each league (see further details below). However, we believe that our check was accurate and informative enough to indicate TM’s actual level of precision.

*National team statistics.* Early detection of discrepancies between TM data and federation statistics led us to collect further data from specific nations where the latter information was available. In the table below, we provide an overview of deviations between the two sources for various national teams, based on descriptive statistics indicating the number of appearances made by players as well as the count of how many players that are listed with appearances. For differences in the number of games listed (“Numerical discrepancies”), we show the mean discrepancy across all players, the maximum amount of deviance we found for a single player, and then the standard deviation across all players. In the far-right column, we report on “Nominal discrepancies”, namely the difference between how many players were listed with one or more appearances for the various teams, according to the two different sources, with both absolute discrepancy (i.e., how many players were listed with appearances according to one source, but not the other) and relative discrepancy (e.g., percentage deviation; the number of players in discrepancy divided by 1482 (the total sample size)). For all the measures of discrepancy, we subtracted the TM information from the federation information. Thus, any negative discrepancy value would indicate that players had been listed as having less team experience in the TM data compared to the federation web sites, and vice versa if the discrepancy value is positive. The U23 teams (often used in the Olympic Games) were omitted from our check, because we were unable to collect data for this team category for some of the countries in our sample.

|  | **Numerical discrepancies** | | | **Nominal discrepancies** |
| --- | --- | --- | --- | --- |
| **Team** | **Mean discrepancy** | **Max. discrepancy** | ***SD*** | **Count discrepancy (% of total sample)** |
| **U15** | -0.21 | -10 | 0.93 | -110 (7.42%) |
| **U16** | -0.90 | -20 | 2.54 | -216 (14.57%) |
| **U17** | -0.58 | -20 | 2.54 | -190 (12.82%) |
| **U18** | -0.37 | 16 | 1.63 | -136 (9.18%) |
| **U19** | -0.12 | -16 | 1.20 | -77 (5.20%) |
| **U20** | 0.02 | -10 | 0.78 | -6 (0.40%) |
| **U21** | -0.06 | 7 | 0.55 | -18 (1.21%) |
| **Sr.** | <0.01 | 2 | 0.13 | 3 (0.20%) |

As the negative values for mean numerical discrepancy and count discrepancy show, the TM database tended to underreport U-team appearances, compared to the federation databases. This is likely due to a lack of consistent updating of the TM player profiles. From the category U16 and upwards, the TM database became increasingly more precise, as reflected in the decreasing SD and max. discrepancy values. At the senior level, the TM database is relatively well-aligned with the data provided by the federations. This fits with our general impression of the TM database and other online data sources: player profiles appeared to be completer and more precise for players with experience at higher levels – for example, those who had made appearances in a senior national team – compared to less-accomplished players who had, for instance, only U-team experience. Our study depended on precise U-team statistics, and we regarded the federation data as “completer” and more trustworthy. For this reason, we decided not to use TM’s national team data in any analyses.

*League statistics*. We tested the reliability of the TM data for the top tiers in the 15 highest ranked nations on the UEFA country coefficients list from the 2020/2021 season:

1. Premier League (England)
2. La Liga (Spain)
3. Serie A (Italy)
4. Bundesliga (Germany)
5. Ligue 1 (France)
6. Liga Bwin (Portugal)
7. Eredivisie (Netherlands)
8. Premier Liga (Russia)
9. Jupiler Pro League (Belgium)
10. Bundesliga (Austria)
11. Premiership (Scotland)
12. Premier Liga (Ukraine)
13. Süper Lig (Turkey)
14. Superligaen (Denmark)
15. Protathlima Cyta (Cyprus)

We also examined two major European tournaments: the Champions League (CL) and the Europa League (EL). We spent a comparable amount of time analysing each league: a minimum of approximately 30 minutes (except for the Ukrainian Premier Liga, as we had only one player listed with appearances in this league). We conducted each league check by alternating between players with, and without, listed appearances in the given league. Each player checked was investigated using

1) a re-check on TM

2) Wikipedia (English version)

3) other online sources (e.g., www.altomfotball.no)

Ideally, we hoped to employ official data sources that we could use as points of comparison for the TM data, as we had been able to do for the national team data that we accessed from federation web sites. However, we were unable to find official databases for all the leagues in question. While Wikipedia and other public databases are not ideal sources, they did provide a useful source of reference.

Nevertheless, in several cases, Wikipedia left us unsure about the number of appearances that players had had for leagues, as many player profiles contained unclear information. It was sometimes difficult to distinguish between regular season games on the one hand, and playoff games, relegation games and qualification games on the other. The TM data clearly distinguishes between these types of games, but Wikipedia and other sources listed all such games under the broader category of appearances in each league. To ensure that we were comparing like for like, we counted regular league games only (often by manually subtracting the count of regular league games from the other games).

When we were reasonably certain that the information we were able to gather from Wikipedia was trustworthy and that the number of appearances differed from the information TM provided for the players in questions, we examined this discrepancy more closely. In many cases, a third source was used to confirm the TM estimates. However, in the few cases in which Wikipedia seemed correct, we examined which specific games had been the cause of such deviations. Only in the cases where we were able to document which specific games had caused a discrepancy (e.g., games had been included in one source, but had been left out of another) were we able to suggest that the discrepancy indicated a potential error in the TM data.

Overall, the results of this reliability check suggested that the TM data had a high level of accuracy. For CL, EL, and the top ten tiers in Europe, we were unable to find discrepancies that indicated that there was error in TM’s data. We therefore regarded TM data for the leagues ranked 10 or higher, as well as CL and EL, as correct and satisfactory for our research purposes. We found only minor discrepancies for two players during our check that we believe are due to errors in TM’s estimates. These discrepancies related to appearances in the Scottish Premiership. In both these instances, a player was listed with three fewer games in the Scottish Premiership on TM, compared to Wikipedia and to www.altomfotball.no. The discrepancies were 10 (TM) vs. 13 (Wikipedia and www.altomfotball.no) games, and 30 (TM) vs. 33 (Wikipedia and www.altomfotball.no) games, respectively. The potential errors we found were therefore relatively few and minor. This adds to the findings of Güllich (1), who reported a .99 correlation between TM and Wikipedia statistics for professional players’ youth academy involvement, suggesting that TM’s accuracy is satisfactory for many research purposes.

Based on the similarity between TM and other data sources, we regarded TM as a reliable data source for our numerical variables (i.e., number of appearances), at least for the major tournaments (CL/EL) and top ten tiers in Europe. We were even more confident about the nominal data extracted from TM, which indicated whether a player had had one or more appearances in these competitions, as such data would not be affected by any minor numerical discrepancies. We hope that reliability checks like ours will be useful in future research and become a standard part of studies that use archival online data. It was indeed informative for us ahead of the next step in our analyses, namely our factor analysis.

**References**

1. Güllich A. Selection, De-Selection and Progression in German Football Talent Promotion. *European Journal of Sport Science* (2014) 14(6):530-7. doi: 10.1080/17461391.2013.858371.
